# Difference in plasticity of resting metabolic rate – the proximate explanation to different niche breadth in sympatric *Ficedula* flycatchers

**DOI:** 10.1002/ece3.3987

**Published:** 2018-04-14

**Authors:** S. Eryn McFarlane, Murielle Ålund, Päivi M. Sirkiä, Anna Qvarnström

**Affiliations:** ^1^ Animal Ecology/Ecology and Genetics Evolutionary Biology Centre Uppsala University Uppsala Sweden; ^2^ Finnish Museum of Natural History Zoology Unit University of Helsinki Helsinki Finland; ^3^ Section of Ecology Department of Biology University of Turku Turku Finland; ^4^ Present address: Institute of Evolutionary Biology University of Edinburgh Edinburgh UK

**Keywords:** cross‐fostering, *Ficedula* flycatchers, plasticity, resting metabolic rate

## Abstract

Variation in relative fitness of competing recently formed species across heterogeneous environments promotes coexistence. However, the physiological traits mediating such variation in relative fitness have rarely been identified. Resting metabolic rate (RMR) is tightly associated with life history strategies, thermoregulation, diet use, and inhabited latitude and could therefore moderate differences in fitness responses to fluctuations in local environments, particularly when species have adapted to different climates in allopatry. We work in a long‐term study of collared (*Ficedula albicollis)* and pied flycatchers (*Ficedula hypoleuca*) in a recent hybrid zone located on the Swedish island of Öland in the Baltic Sea. Here, we explore whether differences in RMR match changes in relative performance of growing flycatcher nestlings across environmental conditions using an experimental approach. The fitness of pied flycatchers has previously been shown to be less sensitive to the mismatch between the peak in food abundance and nestling growth among late breeders. Here, we find that pied flycatcher nestlings have lower RMR in response to higher ambient temperatures (associated with low food availability). We also find that experimentally relaxed nestling competition is associated with an increased RMR in this species. In contrast, collared flycatcher nestlings did not vary their RMR in response to these environmental factors. Our results suggest that a more flexible nestling RMR in pied flycatchers is responsible for the better adaptation of pied flycatchers to the typical seasonal changes in food availability experienced in this hybrid zone. Generally, subtle physiological differences that have evolved when species were in allopatry may play an important role to patterns of competition, coexistence, or displacements between closely related species in secondary contact.

## 1 INTRODUCTION

A major predicted response to climate change is that species may expand or contract their distribution ranges (e.g., Tayleur et al., 2015). Many recently genetically diverged species will therefore come into secondary contact, after a period of allopatry, when they still have overlapping niche requirements. As a consequence, there may be strong competition over limited resources and in the in the worst‐case scenario, competitive exclusion can occur (Pigot & Tobias, 2013). Theoretical models suggest that competitive exclusion can be avoided when there is environmental heterogeneity favoring different species in different times or space (Amarasekare & Nisbet, 2001; Chesson & Warner, 1981; Chesson & Huntly, 1997). However, it is expected that at least some divergence in niche use between young species is required to cause such different fitness responses across heterogeneous environments, which allow for coexistence. The identification of key characteristics that underlay differences in niche requirements of young species is therefore important if we want to understand and predict how biodiversity in communities will be affected when species come into secondary contact.

Physiological differences, such as those resulting from divergent climate adaptation in allopatry, are underestimated in the context of facilitating initial coexistence of young species at secondary contact and in relation to setting the stage for processes of ecological and reproductive character displacements (Keller & Seehausen, 2012; Qvarnström, Ålund, McFarlane, & Sirkiä, 2016). This omission probably reflects practical limitations faced by empirical researchers as physiological traits cannot be measured on dead animals and plants in museum collections. Furthermore, when measured in live organisms, understanding physiological mechanisms may require more invasive methods than when measuring morphological traits. However, even early researchers such as Darwin (1859) and Wallace (1878) realized that ecological adaptations in the temperate zone are largely driven by climate, which means that even comparatively short periods of allopatry may result in divergence in traits underlying such adaptations between populations.

Adaptation to different climates can be seen as physiological tolerance of environmental factors such as temperature or moisture (Somero, 2010). Variation in climate also strongly influences selection acting on life history traits (e.g., growth and developmental rates; Lourdais, Shine, Bonnet, Guillon, & Naulleau, 2004) and trophic interactions and their timing (i.e., availability of prey, predators, and parasites; Stenseth et al., 2002). For example, insectivorous birds breeding in the temporal zone need to time the hatching of their broods so that the nestling growing period is in synchrony with the highest abundance of nutrient‐rich insects such as caterpillar larvae (Both et al., 2004; Visser, Van Noordwijk, Tinbergen, & Lessells, 1998). The abundance of these insects in turn depends on plant phenology that is tightly associated with climate, including temperature and latitude. Selection patterns acting on the traits controlling the ability of insectivorous birds to either time their breeding appropriately, or cope with variable food availability while feeding their offspring, are therefore expected to vary with climate.

One physiological trait that likely plays a central role when organisms adapt to different climate conditions is resting metabolic rate (RMR), “the energetic cost of self‐maintenance” (Burton, Killen, Armstrong, & Metcalfe, 2011). This is because RMR is related to thermoregulation (Klaassen, Oltrogge, & Trost, 2004; Naya, Spangenberg, Naya, & Bozinovic, 2013) and is also known to covary with attributes of the habitat individuals, populations, or species live in, including latitude (Lovegrove, 2003), climate (Song & Wang, 2006), and/or productivity (Mueller & Diamond, 2001). RMR is a somewhat phenotypically plastic trait that allows organisms to respond to changes in their environment. For examples, guppies (*Poecilia reticulata*) change their RMR in response to predators (Handelsman et al., 2013), mouse (*Mus musculus*) RMR fluctuates over the course of pregnancy (Speakman & McQueenie, 1996), and generally, birds’ RMR changes in response to cool, seasonal temperatures (McKechnie, 2008). In addition, experimental studies in hamsters (*Phodopus sungorus*) have demonstrated marked differences in metabolic rate in response to short‐term differences in ambient temperature (Boratyński, Jefimow, & Wojciechowski, 2016, 2017). Thus, there is ample evidence suggesting that plasticity in metabolic rate allows organism to quickly adjust to short‐term changes in the environment. By extension, both the mean and the degree of individual plasticity in RMR among organism in a population can be expected to vary across environments. Differences in environmental conditions experienced should impose distinct differences in the selective regimes acting on this physiological trait and lead to responses in RMR. Given that metabolic rate is a trait closely linked to life history variation (Burton et al., 2011) and climate adaptation, this trait is likely to be affected by differences in ecological environments experienced in the recent past and to affect future evolutionary trajectories at secondary contact.

Collared (*Ficedula albicollis*) and pied (*Ficedula hypoleuca*) flycatchers are closely related insectivorous passerines that co‐occur in central Europe and on the Swedish islands of Öland and Gotland, where they regularly form mixed species pairs (Cramer, Ålund, McFarlane, Johnsen, & Qvarnström, 2016), prefer the same nesting sites (Lundberg & Alatalo, 1992), and prey items (Wiley et al., 2007). These two species diverged less than 1 million years ago, probably mainly during allopatric conditions but with repeated instances of secondary contact and hybridization (Nadachowska‐Brzyska et al., 2013). Collared flycatchers have only recently (i.e., during the early 1960s) settled on Öland, which makes this system ideal to study recent secondary contact after a period of allopatry (Qvarnström et al., 2016; Qvarnström, Rice, & Ellegren, 2010). Collared flycatchers have quickly displaced pied flycatchers from the preferred breeding sites on Öland (Vallin, Rice, Arntsen, Kulma, & Qvarnström, 2012), but pied flycatchers are better able to breed in less preferred poorer habitats where the levels of caterpillar biomass are lower (Sirkiä et al., 2017). As a consequence, when pied flycatchers are pushed from good habitats, they can both escape competition from collared flycatchers and reduce the risk of hybridization by breeding in poorer habitats (Rybinski et al., 2016). Further, collared flycatchers face stronger selection to breed earlier in the year to match the peak of caterpillar availability (both species’ preferred food source), leading to temporal isolation (Sirkiä et al., 2017). It is clearly better for collared flycatchers to breed as early as possible in the breeding season to match this food peak, while pied flycatchers can successfully breed later in the season, and in this way, avoid hybridization (Sirkiä et al., 2017). The two species appear to be very similar ecologically, and the underlying traits that make pied flycatchers able to utilize a broader habitat and temporal niche than collared flycatchers are yet unknown. Based on experimental swapping of offspring between heterospecific nests, we were able to establish that this crucial difference in niche requirement between the two species is at least partly due to intrinsic differences between the nestlings (Qvarnström, Svedin, Wiley, Veen, & Gustafsson, 2005; Qvarnström, Wiley, Svedin, & Vallin, 2009). Specifically, nestling pied flycatchers are less likely to die from starvation during lower levels of food availability (i.e., pied flycatcher nestlings are “hardier”; Qvarnström et al., 2005).

The aim of this study was to examine whether there is an intrinsic difference in RMR between the nestlings of the two species that could provide a proximate explanation for why pied flycatcher nestlings are less sensitive to lower levels of food availability (i.e., in poorer habitats late in the season). We predict that pied flycatchers either have a generally lower RMR, demanding less food, or, that they have the ability to adjust their RMR to fluctuations in food availability. The main aim of this study was to experimentally test these predictions using a cross‐fostering design to control for genetic effects while varying the environment. Specifically, we investigated how nestling RMR responded to (1) natural fluctuations in food availability estimated as temperature, which indicates the parents’ onset of breeding in relation to the progress of the spring through the degree of match with the temporal peak in food abundance, and (2) experimentally changed brood size, which influenced individual nestlings access to food through affects on nestling competition.

## 2 METHODS

To isolate variation in intrinsic differences between nestlings of the two species and to remove effects of parental territory, quality, and behavior, we performed a reciprocal, cross‐fostering experiment on a monitored mixed population of nestbox–breeding collared and pied flycatchers on Öland (57°10N, 16°58E), Sweden in 2013, 2014, and 2015. In these years, in the general population, there was no difference in clutch size between collared and pied flycatchers (collared flycatchers mean clutch size = 6.49 ± 0.7 (2013), 6.70 ± 0.8 (2014), 6.52 ± 0.7 (2015), pied flycatchers mean clutch size = 6.51 ± 0.8 (2013), 6.48 ± 0.8 (2014), 6.81 ± 0.7 (2015)). We matched nests based on species, laying date, brood size, and average nestling mass, to ensure that nestling growth would not be affected by initial biases between the cross‐fostered broods (Hadfield, Heap, Bayer, Mittell, & Crouch, 2013). The vast majority of swaps were between nests of the same brood size, although some broods differed by one nestling, and one swap differed by two. We moved two nestlings from each brood between the matched nests. If the nests did not fully overlap in nestling size (i.e., did not have approximately the same mean nestling mass), we moved the two largest nestlings from the lighter brood to the heavier brood, and vice versa. We moved nestlings when they were 3 days old, since nestling mass at 3 days old does not predict nestling mass at 12 days old (Qvarnström et al., 2005). All nestlings were individually marked using toenail clipping (Qvarnström et al., 2009), which allowed us to identify them individually when they were 6 days old, at which point they were ring‐marked with standard alphanumeric rings. Nestlings were weighed when 3, 6, and 12 days old. This allowed us to measure nestling growth rate as the difference between 12‐day mass and 3‐day mass divided by 9 days of growth. This assumes a linear growth rate, and essentially allows us to look at the nestling mass increase after cross‐fostering.

### 2.1 Environmental heterogeneity

Collared flycatcher nestlings tend to have lower growth and survival rates than pied flycatchers when their parents breed relatively late in the season (Qvarnström et al., 2005, 2009). We used mean ambient temperature during the nestling period (between day 3 and day 12 posthatching) as an indication of the quality of the nestling environment (i.e., food availability) resulting from variation in the parents’ onset of breeding in relation to the progress of the spring. We accessed weather data from the Swedish meteorological and hydrological institute, SMHI (http://opendata-download-metobs.smhi.se/explore/). SMHI has two weather stations close to our study areas on both Öland and in Kalmar. All nestboxes were within 25 km of a weather station, and 95% of boxes were less than 20 km away. We used mean temperature estimates from the nearest weather stations from when the nestlings were 3 days old until when they were 12 days old (i.e., after the cross‐fostering), computed separately for each experimental nest. We refer to this mean environmental temperature as “ambient temperature.” We used ambient temperature as a proxy for food availability because it has been used as a proxy in other studies (Both, 2010). However, ambient temperature correlates with food availability at our field site, where a warm temperature during the nestling growth period corresponds to an advanced stage of the spring and low abundance of nutrient‐rich caterpillar larvae as the peak of caterpillar larvae coincide with an early stage of tree leaf growth. Caterpillar feces (frass) estimates in 2013 and 2014 show that, after accounting for differences among years and tree species, there is a strong negative correlation between caterpillar availability and ambient temperature during the month of June (*F*_1,500.5 _= 9.23, *p* = .0025, *R* ^2 ^= .139). Collection of frass in this system is described in detail in (Rybinski et al., 2016). As the development of tree leaves and the growth of caterpillars that eat these leaves depend on the ambient temperature rather than on dates per se, we included ambient temperature rather than laying date (with which temperature is confounded) in our analyses.

In order to experimentally affect the rearing environment, we performed brood size manipulations where we moved four nestlings from one nest into a matched nest, and two nestlings from the matched nest to the original nest in 2014 and 2015. Brood enlargement increases competition between nestlings and reduces relative food availability for each nestling. The logic behind this experimental design is that one nest in the matched pair has two extra nestlings, and one has two fewer, but both have a mix of nestlings from both nests, allowing us to look at environmental effects while controlling for potential genetic effects. Additionally, we did standard reciprocal cross‐fostering in 2014 and 2015 to have control nests (as was done in 2013), which retained the original number of nestlings. In total, we had 148 experimental nests but 33 were predated or died for other reasons (e.g., poor weather conditions) prior to 8 days posthatching, resulting in 115 remaining experimental nests across 3 years. Experimental sample sizes are outlined in Table 1. Finally, we measured nestling mass when nestlings were 8 days old to the nearest 0.1 g using a Pesola balance.

**Table 1:** Year, species, and treatment specific sample sizes of wild 8‐day‐old collared and pied flycatcher nestlings in which resting metabolic rate was measured

Year	Species	Control	Enlargement	Reduction
2013	Collared Flycatcher	26	NA	NA
	Pied Flycatcher	38	NA	NA
2014	Collared Flycatcher	25	17	20
	Pied Flycatcher	8	7	7
2015	Collared Flycatcher	25	5	7
	Pied Flycatcher	8	5	1

### 2.2 Metabolic rate

We measured RMR of 8‐day‐old nestlings using a custom respirometer. We aimed to take one original and one foster nestling from each experimental nest overnight to the laboratory. Nestlings were taken after 6 p.m. at night and returned to their nest before 5:30 a.m. the next morning to avoid disturbing the parental feeding schedule. While being measured in the respirometer, nestlings were in a climate cabinet set at 28°C (hereafter climate controlled temperature) to ensure that they were in their thermoneutral zone (Bushuev, Husby, Sternberg, & Grinkov, 2012; Lasiewski, Hubbard, & Moberly, 1964). To measure the rate of oxygen consumption, carbon dioxide production, and water vapor production for each nestling, we used an FMS respirometer, RM‐8 multiplexer, PP‐2H field pump, and FlowBar‐8 (Sable Systems, Henderson, NV, USA). The respirometer was regularly calibrated, using dry outside air run through a mix of ascarite and dririte to calibrate the oxygen monitor to 20.95%. The humidity monitor was span calibrated after running outside air through magnesium perchlorate until the air was dry. The CO_2_ monitor was span calibrated using a known concentration of 0.5% CO_2_ in nitrogen. All calibrations were performed according to the manufacture's recommendation. This respirometry set up allowed us to measure seven birds at a time, with a reference chamber. Nestlings were placed in an individual glass chamber (8 cm by 30 cm) for 4 hr; each chamber was measured six times for five minutes, and since we were able to run the respirometer twice per night, we could measure 14 birds each night, between either 8 p.m. until 12 a.m. or 12 a.m. until 4 a.m. Chamber and cycle assignment was performed haphazardly. We used a flow rate of approximately 400 ml/min, consistent with previous studies of passerines of a similar size (Broggi et al., 2007; Lewden, Petit, & Vézina, 2012; Rønning, Jensen, Moe, & Bech, 2007; Versteegh, Helm, Dingemanse, & Tieleman, 2008).

We analyzed each measure of RMR using the manufacturer's software, Expedata. We used macros in Expedata to extract readings from the respirometer and compare to baseline. The macros also accounted for a lag when the multiplexer changed between chambers (Sable Systems International, Las Vegas, USA). We judged this lag by eye and applied it equally to all files. We measured oxygen levels (O_2_), carbon dioxide (CO_2_), and water vapor pressure and used equations 9.3 and 9.4 in Lighton (Lighton, 2008) to calculate the rate of oxygen consumption (VO_2_), as RMR. As we measured metabolic rate six times for each bird, we needed to decide on the most representative measurement. We always discarded the first measurement to allow the bird to settle down. From the remaining measurements, we chose the one with the lowest standard deviation, as this lack of variation was assumed to be indicative that the animal was very still or sleeping (Lighton, 2008). Further, we removed those estimates of RMR that were 2 standard deviations away from the mean (seven estimates were removed as they were more than 2 standard deviations above the mean), as we assumed that these birds were not sleeping.

### 2.3 Statistical methods

Our first aim was to determine if the brood manipulation experiment had affected growth rate as expected. To do this, we fitted a linear mixed effects model with growth rate as the response variable and included foster status, treatment, species and year as fixed effects, and nest of origin, as well as foster nest as random effects. We also included an interaction between species and treatment to determine if the two species responded differently to the experiment.

Our second aim was to test whether there was a consistent difference in RMR between collared and pied flycatcher nestlings across environmental conditions. To do this, we used a linear mixed effects model with RMR as a response variable, year, mass, species, and the interaction between mass and species as fixed effects and foster nest and nest of origin as random effects. Our third aim was to investigate whether the RMR of nestlings of the two species responded differently to natural variation in ambient temperature and to an experimental brood manipulation. We used linear mixed effects models that included ambient temperature and brood manipulation separately, in models that also included year, species, mass, and interactions with both mass and species as fixed effects. We tested these in separate models to avoid over fitting any one model. Thus, all models explicitly tested three‐way interactions (i.e., mass × species × environmental variable), although we recognize that these environmental variables tested in different models may explain some of the same variance. We also included foster nest and nest of origin as random effects to account for nonindependence in all models. For all models except for the model assessing an effect of brood manipulation, we used only control nestlings (i.e., those that that were in experimental cross‐foster nests, but not brood enlargement or reduction nests), so as to not need to account for the experimental effects while testing other factors. To test for an effect of food availability, we included control nestlings in addition to nestlings that were part of the brood size experiment.

We report analysis of variance (ANOVA) comparisons below, and all estimates, standard errors, *t*‐values, and *p*‐values and false discovery rate adjusted *p*‐values to account for multiple testing (Forstmeier & Schielzeth, 2011) in Tables 2, 3, 4 and, we report both *p*‐values in the main text. All ANOVAs were type 3 sums of squares. We did all analyses using the lme4 package in R (Bates, Maechler, Bolker, & Walker, 2014; R Core Team, 2013). We estimated the degrees of freedom using a Satterthwaite approximation and assessed significance of the fixed effects in the mixed effects models using the lmerTest package (Kuznetsova, Brockhoff, & Christensen, 2014). We estimated marginal *R* ^2^ for all models as described by (Nakagawa & Schielzeth, 2013). Using a Kenward–Roger estimation of degrees of freedom gave similar results. All model assumptions were met as determined by a visual assessment of the model residuals.

**Table 2:** We used a mixed effects model to compare the resting metabolic rate of collared and pied flycatcher nestlings. We fit year, mass, and species as fixed factors, and an interaction between the mass and species. We additionally fit foster nest and nest of origin as random effects. We did not find evidence of an interaction between species and mass. Bold values refer to effects that were significant before accounting for multiple testing

Fixed effects	Estimate	Std. Error	*df*	*t* value	*p* Value	Adjusted *p* value
(Intercept)	0.087	0.60	115.4	0.15	.884	.976
Species (pied flycatchers)	0.056	1.02	103.2	0.06	**.**956	.976
Mass	0.133	0.04	115.2	3.00	**.003**	.073
Year (2014)	−0.196	0.15	79.3	−1.34	.185	.347
Year (2015)	−0.220	0.15	80.4	−1.49	.141	.303
Mass × Species (pied flycatchers)	−0.013	0.08	104.6	−0.17	.866	.976

Random effects	Variance					
Foster nest	0.114					
Nest of Origin	0.051					
Residual	0.133					

**Table 3:** We used a mixed effects model to compare RMR between collared and pied nestlings measured on Öland between 2013 and 2015. We fit year, ambient temperature (°C) during the nestling growth phase, species and an interaction between the two as fixed effects and foster nest and nest of origin as random effects. We found a significant interaction between ambient temperature and species. The contrast species was always collared flycatchers, where the estimates including species are always the difference in pied flycatcher nestlings. Bold values refer to effects that were significant before accounting for multiple testing

Fixed effects	Estimate	Std. Error	*df*	*t* value	*p* Value	Adjusted *p* value
(Intercept)	−23.713	13.83	109.9	−1.72	.089	.223
Ambient temperature	1.574	0.91	110.2	1.73	.087	.223
Species (Pied Flycatchers)	58.548	26.91	110.8	2.18	**.032**	.159
Mass	1.933	1.04	105.9	1.87	.065	.216
Year (2014)	−0.182	0.20	83.6	−0.93	.355	.592
Year (2015)	−0.241	0.18	81.9	−1.34	.183	.347
Temperature × Species (pied flycatchers)	−3.732	1.70	110.6	−2.20	**.030**	.159
Temperature × Mass	−0.119	0.07	106.1	−1.74	0.085	.223
Species (pied flycatchers) × Mass	−4.210	2.09	111.9	−2.01	**.046**	.174
Temperature × Species (pied flycatchers) × Mass	0.268	0.13	112.1	2.03	**.045**	.174

Random effects	Variance					
Foster nest	0.120					
Nest of Origin	0.057					
Residual	0.119					

**Table 4:** We used a mixed effects model to compare RMR between 8‐day‐old collared and pied flycatcher nestlings on Öland between 2013 and 2015. We fit year, the treatment from a brood manipulation experiment (enlargement, reduction, control), species and mass and all interactions between treatment, species and mass as fixed effects, and nest of origin and foster nest as random effects. The contrast species was always collared flycatchers, where the estimates including species are always the difference in pied flycatcher nestlings. The contrast treatment was control, thus the estimates for enlargement and reduction treatments are in comparison with the control treatment. Bold values refer to effects that were significant before accounting for multiple testing

Fixed effects	Estimate	Std. Error	*df*	*t* value	*p* Value	Adjusted *p* value
(Intercept)	0.020	0.65	169.9	0.03	.976	.975
Enlargement	−0.963	1.23	135.3	−0.78	.436	.688
Reduction	−0.328	1.31	169.2	−0.25	.803	.976
Species (Pied Flycatchers)	0.232	1.11	163.4	0.21	.835	.976
Mass	0.138	0.05	170.6	2.85	**.004**	.073
Year (2014)	−0.232	0.15	105.1	−1.58	.118	.273
Year (2015)	−0.177	0.15	106.3	−1.20	.235	.414
Enlargement × Species (pied flycatchers)	−1.401	2.88	172.8	−0.49	.628	.856
Reduction × Species (pied flycatchers)	−7.923	3.54	173.4	−2.24	**.027**	.159
Enlargement × Mass	0.061	0.10	135.0	0.63	.530	.796
Reduction × Mass	0.007	0.10	171.8	0.07	.947	.976
Species (pied flycatchers) × Mass	−0.027	0.09	164.5	−0.31	.760	.976
Enlargement × Species (pied flycatchers) × Mass	0.129	0.24	173.9	0.54	.588	.840
Reduction × Species (pied flycatchers) × Mass	0.669	0.28	172.2	2.42	**.017**	.157

Random effects	Variance					
Foster nest	0.121					
Nest of origin	0.063					
Residual	0.173					

## 3 RESULTS

We weighed 831 nestlings at 3 days old in 2013, 2014, and 2015, and cross‐fostered 321 nestlings between nests. We found that collared flycatcher nestlings that survived to 12 days old gained an average of 0.971 ± 0.2 g/day and pied flycatcher nestlings gained an average of 0.948 ± 0.2 g/day. We found no effect of foster status on growth rate (*F*_1,423.5 _= 0.571, *p* = .450, Table 5), we did find an effect of brood manipulation on growth rate (*F*_1,97.4 _= 3.912, *p* = .0232, Table 5), but we did not find a significant interaction between species and brood enlargement on growth rate (*F*_2,194.6 _= 0.553, *p* = .576, Table 5). Taken together, species, year, fostering status, brood manipulation, and an interaction between species and brood manipulation explained 18.2% of the variation in nestling growth rate.

**Table 5:** We used a mixed effects model to compare the growth rate of collared and pied flycatcher nestlings. We fit foster status, brood enlargement experimental treatment and species as fixed factors, and an interaction between treatment and species. We additionally fit foster nest and nest of origin as random effects. Bold values indicate significance

Fixed effects	Estimate	Std. Error	*df*	*t* value	*p* Value
(Intercept)	1.058	0.03	127.3	30.31	<2e‐16
Non‐fostered nestling	0.007	0.01	423.5	0.76	.450
Enlargement	−0.146	0.05	111.6	−2.93	**.004**
Reduction	−0.030	0.05	107.7	−0.59	.560
Species (pied flycatchers)	−0.051	0.04	129.5	−1.28	.205
Year (2014)	−0.106	0.05	115.1	−2.27	**.025**
Year (2015)	−0.106	0.04	112.3	−2.38	**.019**
Enlargement × Species (pied flycatchers)	0.061	0.07	173.8	0.83	.409
Reduction × Species (pied flycatchers)	0.082	0.09	200.9	0.93	.354

Random effects	Variance				
Foster nest	0.018				
Nest of origin	0.005				
Residual	0.010				

We measured the RMR of 64, 84, and 51 nestlings in 2013, 2014, and 2015 (see species‐specific sample sizes in Table 1). We found that 8‐day‐old collared flycatcher nestlings had an average mass of 12.95 ± 1.6, and average RMR of 1.54 ± 0.7 ml/min and that 8‐day‐old pied flycatcher nestlings had an average mass of 12.53 ± 1.1 and average RMR of 1.58 ± 0.6 ml/min. There was no significant interaction between species and mass on RMR (*F*_1,104.6 _= 0.028, *p* = .866, *p*_adj _= .921; Table 2), nor a mass independent effect of species on RMR (*F*_1,103.2 _= 0.031, *p* = .957, *p*_adj _= .956). Mass, species, and the interaction between mass and species explained 14.2% of the variation in RMR.

**Figure 1:**
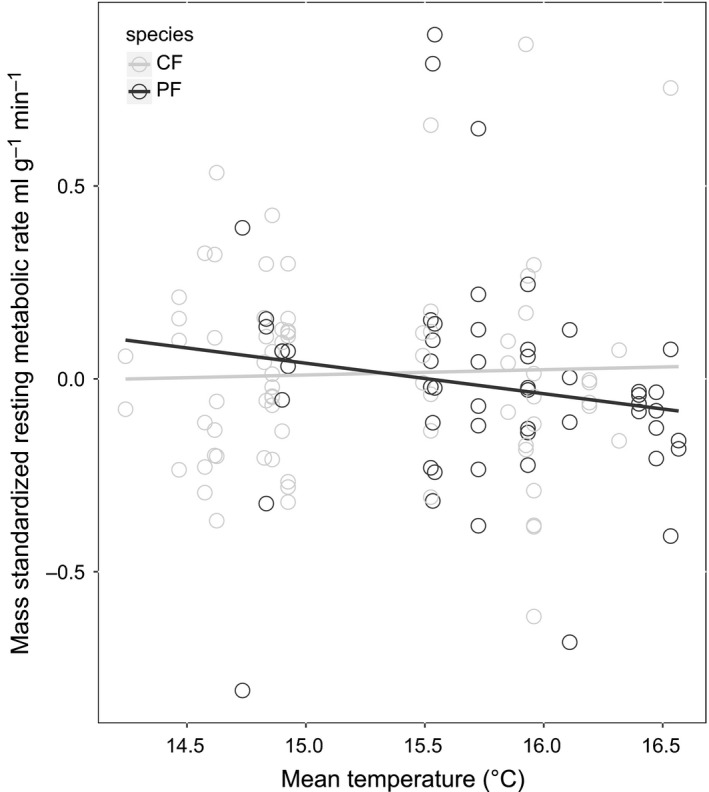
The relationship between resting metabolic rate (RMR) and ambient temperature (as an indicator of food availability) in nestling collared (CF) and pied (PF) flycatchers. A high ambient temperature is associated with relatively low abundance of caterpillar larvae at our study sites. Metabolic rate is displayed as a residual of metabolic rate regressed against nestling mass and year. We found an association between pied flycatcher nestling RMR and mean ambient temperature during the nestling growth stage that was not present in collared flycatcher nestlings

We examined whether the covariance between nestling RMR and environmental factors differed between the two species. Specifically, we first tested whether there were differences in RMR between the two species that could be explained by natural variation in mean ambient temperature during the growth period, which we used as a proxy for food availability. There was a significant interaction between ambient temperature, species, and mass explaining variation in RMR (*F*_1,112.1 _= 4.109, *p* = .045, *p*_adj _= .114, Figure 1, Table 3). Together, ambient temperature, mass, species, and the associated interactions explained 19.0% of the variation in RMR. Particularly in heavier nestlings, pied flycatcher RMR was lower in higher ambient temperatures, but no similar pattern was found in collared flycatchers.

**Figure 2:**
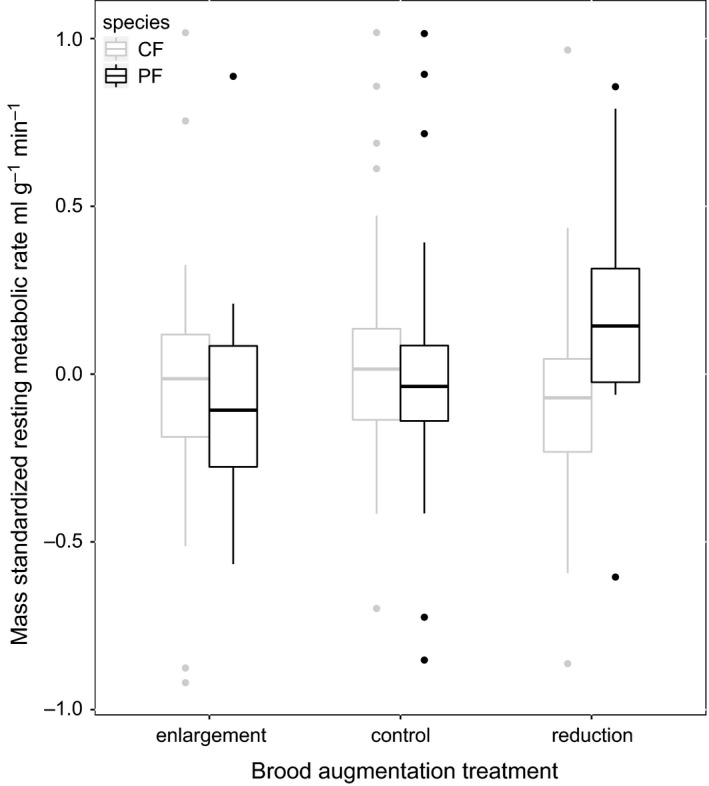
Comparison of the effects of brood size manipulation on RMR in nestling collared and pied flycatchers. Metabolic rate displayed as a residual where metabolic rate has been regressed against mass and year. Nests were matched by hatching date, number of nestlings and mean nestling mass, and nestlings were swapped between nests. Control nests had two nestlings swapped between them so that each nest had two foster siblings in it. Enlargement nests were paired with reduction nests, where four nestlings were taken from a reduction nest and swapped with two from an enlargement nest. In this way, enlargement nests had four foster siblings, and two more nestlings than originally, and reduction nests had two foster siblings and two fewer nestlings than originally had hatched in the nest. We found an interaction between reduced brood size and mass on RMR in pied flycatchers when compared to control broods, although we found no significant difference in how the two species reacted to being in larger broods, and therefore only a marginally significant effect of the experimental treatment

We examined whether there was any difference in how the nestlings of the two species adjusted their RMR in relation to an experimental manipulation of the brood environment. We found a marginal effect of the brood manipulation experiment over all (*F*_2,156.1 _= 3.20, *p* = .044, *p*_adj _= .114), and a marginal interaction between treatment, species, and mass as we might have expected (*F*_2,153.8 _= 2.92, *p* = .057, *p*_adj _= .114). Examination of specific contrasts showed that this relationship was driven by a difference between pied flycatcher nestlings in reduced nests and those in control nests, with an interaction with mass (Est = 0.669 ± 0.3, *df* = 172.2, *t* = 2.42, *p* = .017, *p*_adj _= .159, Table 4) although we found no difference in how the two species reacted to being in larger broods (Table 4, Figure 2). All together, brood manipulation treatment, mass, species, the interactions between these and a covariate of year explained 20.1% of the variation in RMR.

## 4 DISCUSSION

We compared how natural variation in environmental conditions experienced during nestling growth and an experimental manipulation of the nest environment affected nestling RMR in two competing *Ficedula* flycatcher species. We found that both the relationship between the natural degree of mismatch with the food peak (here estimated as mean ambient temperature during the nestling growth phase) and nestling RMR and the effects of a brood size manipulation on nestling RMR differed between the two species. Pied flycatcher nestling RMR was lower in higher ambient temperatures, when their parents had bred relatively late in relation to the peak in food availability (Figure 1). Because a low metabolic rate generally is thought to be beneficial when food availability is low (Mueller & Diamond, 2001), this reduction in metabolic rate could lower the risk of starvation. In addition, RMR of pied flycatcher nestlings was affected by the brood size manipulation experiment such that a higher RMR was observed in experimentally reduced broods (Figure 2, Table 4). Nestlings are expected to experience less competition over food when they have fewer nestmates (Moreno, Cowie, Sanz, & Williams, 1995; Sanz, 1997) and higher RMR has been related to increased growth rate or survival when food is not limited (i.e., the “increased intake” hypothesis; Kersten & Piersma, 1987; Zub, Borowski, Szafrańska, Wieczorek, & Konarzewski, 2014 but see Swanson, McKechnie, & Vézina, 2017). In contrast, collared flycatcher nestling RMR was not affected by ambient temperature, nor an interaction between temperature and mass. It thus appears that pied flycatchers have a more flexible RMR in response to environmental variables than collared flycatchers do.

### 4.1 Nestling RMR while growing

Differences in RMR might be particularly relevant during periods of exponential growth (i.e., between approximately 3 and 10 days old), such as when we measured it here. The estimates of RMR that we report here are approximately two times as high as those that have been reported for other bird species that are between 10 and 20 g (0.84 ml/min ± 0.2; Stager et al., 2015), and substantially higher than what has previously been reported in pied flycatchers (i.e., 0.608 ml/min; Moreno & Carlson, 1989, 0;.887 ml/min; Bushuev et al., 2012). However, these estimates were all in adult, or nearly fledged nestlings, while our study was on growing nestlings, specifically during the period of nestling exponential growth. Growth is likely to be extremely energy intensive, thus individuals not needing to expend extra energy on growth should be expected to have a lower RMR than growing nestlings. Indeed, it was found that blue tit nestlings only 1 day older had a significantly lower RMR than younger nestlings (Nilsson, Åkesson, & Nilsson, 2009), and the estimates that we report here for nestlings are higher than we have previously reported in male, adult collared and pied flycatchers (collared flycatchers 1.27 ± 0.5, pied flycatchers 1.04 ± 0.4; (McFarlane, Sirkiä, Ålund, & Qvarnström, 2016). Thus, as daily expenditures of energy can easily be three times as high as RMR (Moreno & Carlson, 1989), these estimates may not be strictly “RMR” so much as “RMR plus the cost of growth.”

### 4.2 Flexible nestling RMR and broad niche use in pied flycatchers

To emphasize the importance of the pied flycatcher nestlings response to the environment, we can determine the actual energetic savings of a nestling with a lower RMR compared to one with a higher RMR. The predicted difference in RMR between the first ambient temperature quartile experienced by nestling pied flycatcher nestlings in our study and the third ambient temperature quartile in pied flycatchers is 4.6e‐3 ml g^−1^ min^−1^, which can be translated to 2.18e‐5 kcal for an average sized pied flycatcher nestling (Schmidt‐Nielsen, 1997). For a small, growing songbird, this could be substantial. In a natural environment, where food availability for nestlings is constrained by the ability of the parents to provide it, this extra energy may have resulted in a considerable fitness gain. Thus, a lower RMR may represent an important adaptation to the typical steep decline in food availability generally experienced as the spring is progressing at these northern breeding sites. Future studies could specifically examine whether there is indeed a fitness benefit to parents breeding late in relation to the peak in food abundance when they have offspring with lower RMR s and/or to specific offspring with relatively low RMR when they have hatched late in relation to the peak in food abundance.

The robustness to a mismatch between breeding and peak of food abundance (Qvarnström et al., 2005, 2009; Rybinski et al., 2016) and an adjustment of nestling pied flycatchers’ RMR to their environment (found in the present study) may reflect (1) an adaptive plastic response where parents are able to assess the environment and adjust their nestlings’ RMR accordingly (e.g., through hormones deposited into the eggs), (2) there may be genetic variation correlated to timing of breeding via environmental cues where some individuals have become adapted to comparatively late breeding (and from the offspring perspective, growth late in the season) under warmer conditions with less food or (3) an adaptive plastic response by the nestlings themselves. All these possibilities could explain why pied flycatchers nestlings show a flexible RMR in relation to ambient temperature (Figure 1, Table 3), which may make them robust to variation in food availability. That pied flycatcher nestlings also increased their RMR in response to being experimentally placed with fewer nest mates suggests that the nestlings, at least to some extent, have an intrinsic ability to plastically respond to changes in the environment. Thus, a flexible nestling RMR may be the underlying physiological trait that allows pied flycatcher nestlings to survive better in relatively poor environments, in contrast to collared flycatchers. By extension, this could be a physiological trait subject to ecological character displacement associated with the rapid ongoing niche segregation between the two flycatcher species observed in the Swedish hybrid zone (Rybinski et al., 2016; Sirkiä et al., 2017).

### 4.3 Nonflexible nestling RMR and a narrow niche use in collared flycatchers

In contrast with the patterns found in pied flycatchers, collared flycatcher nestling RMR did not vary with ambient temperature experienced during growth and did not appear to be affected by the brood size manipulation experiment. This difference in plasticity could stem from the narrower climatic niche that collared flycatchers have inhabited before colonizing the Swedish Baltic islands (Qvarnström et al., 2010). The smaller, more southerly distribution of collared flycatchers might have limited selection for a plastic RMR. Generally, habitat and diet specialization are highest at low latitudes and in bird species with smaller distributions (Belmaker & Jetz, 2011). Thus, the difference in plasticity of RMR between collared and pied flycatcher nestlings that we demonstrate here might stem from differences that evolved in allopatry, while collared flycatchers experienced a less variable environment.

An important question then becomes whether nestling RMR will remain less flexible in collared flycatchers as compared to pied flycatchers in the Swedish hybrid zone? Collared flycatchers are under stronger selection to adjust to the changing food peak (Sirkiä et al., 2017). Additionally, while pied flycatchers are displaced into poorer quality habitat (i.e., that habitat with fewer available caterpillars), the quality of the habitat that collared flycatchers breed in has instead slightly increased during the last 15 years (Rybinski et al., 2016). Taken together, the niche use of collared flycatchers appears to be highly conserved and a nonflexible RMR of nestling collared flycatchers appears to be an important physiological trait underlying this somewhat higher degree of niche specialization as compared to the pied flycatchers.

## 5 CONCLUSION

Studies in community ecology have shown that competitive interactions and relative fitness responses of species to environmental variation can drive further niche differentiation (McGill, Enquist, Weiher, & Westoby, 2006; Amarasekare, 2003; Chesson, 2000). However, the physiological properties that set the stage for the outcome of such interactions and for differences in fitness responses to environmental variation often remain unknown. Metabolic rate has been suggested to drive many interactions in ecology (Spicer & Gaston, 2009), but this has yet to be applied to theories of ecological speciation and coexistence of young species pairs. We have reported here that a negative relationship between RMR and ambient temperature experienced during nestling growth found in pied flycatchers, but absent in the competing closely related collared flycatchers, could be a mechanism promoting regional coexistence between these two species. The higher robustness to late breeding (and hence higher ambient temperatures and lower food supply) has been suggested to help pied flycatchers “escape” competition from collared flycatchers and enable ecological and reproductive character displacement (Qvarnström et al., 2009). However, the actual traits subject to ecological character displacement were previously unknown. Here, we provide experimental evidence compatible with RMR being a crucial trait in this context. We argue that the comparatively low RMR of nestling pied flycatchers associated with poor environmental conditions reflects a better adaptation to the local seasonal changes in breeding conditions in this young area of secondary contact (i.e., observed in the species with the longer local history). We base this conclusion on the facts that lower RMR generally is linked to adaptation to lower food supply (Mueller & Diamond, 2001) and that an adaptive reduction in RMR associated with a reduction in food supply could lower the risk of starvation (Swanson et al., 2017). The tight association between the evolution of variation in life history traits across taxa and RMR (Burton et al., 2011) suggests that variation in RMR may play an important role in explaining patterns of coexistence between species, and thus, general patterns of diversity.

## CONFLICT OF INTEREST

None declared.

## AUTHOR CONTRIBUTIONS

SEM and AQ conceptualized the study; SEM, MÅ, and PS performed the fieldwork, including cross‐fostering, and respirometry; SEM analyzed the data; SEM and AQ wrote the manuscript; and all authors revised and approved the final version.

## References

[ece33987-bib-0001] Amarasekare, P. (2003). Competitive coexistence in spatially structured environments: A synthesis. Ecology Letters, 6, 1109–1122. https://doi.org/10.1046/j.1461-0248.2003.00530.x

[ece33987-bib-0002] Amarasekare, P. , & Nisbet, R. M. (2001). Spatial heterogeneity, source‐sink dynamics, and the local coexistence of competing species. The American Naturalist, 158, 572–584. 10.1086/323586 18707352

[ece33987-bib-0003] Bates, D. , Maechler, M. , Bolker, B. , & Walker, S . (2014). lme4: Linear mixed‐effects models using Eigen and S4. R package version 1.1‐7. Retrieved from http://CRAN.R-project.org/package=lme4.

[ece33987-bib-0004] Belmaker, J. , & Jetz, W. (2011). Cross‐scale variation in species richness–environment associations. Global Ecology and Biogeography, 20, 464–474. https://doi.org/10.1111/j.1466-8238.2010.00615.x

[ece33987-bib-0005] Boratyński, J. S. , Jefimow, M. , & Wojciechowski, M. S. (2016). Phenotypic flexibility of energetics in acclimated Siberian hamsters has a narrower scope in winter than in summer. Journal of Comparative Physiology B, 186, 387–402. https://doi.org/10.1007/s00360-016-0959-3 10.1007/s00360-016-0959-3 PMC4791479 26803319

[ece33987-bib-0006] Boratyński, J. S. , Jefimow, M. , & Wojciechowski, M. S. (2017). Individual differences in the phenotypic flexibility of basal metabolic rate in Siberian hamsters are consistent on short‐and long‐term timescales. Physiological and Biochemical Zoology, 90, 139–152. https://doi.org/10.1086/689870 28277958 10.1086/689870

[ece33987-bib-0007] Both, C. (2010). Food availability, mistiming, and climatic change (pp. 129–147). Oxford, UK: Effects of climate change on birds. Oxford University Press.

[ece33987-bib-0008] Both, C. , Artemyev, A. V. , Blaauw, B. , Cowie, R. J. , Dekhuijzen, A. J. , Eeva, T. , … Järvinen, A. (2004). Large–scale geographical variation confirms that climate change causes birds to lay earlier. Proceedings of the Royal Society of London B: Biological Sciences, 271, 1657–1662. https://doi.org/10.1098/rspb.2004.2770 10.1098/rspb.2004.2770 PMC1691776 15306284

[ece33987-bib-0009] Broggi, J. , Hohtola, E. , Koivula, K. , Orell, M. , Thomson, R. , & Nilsson, J. Å. (2007). Sources of variation in winter basal metabolic rate in the great tit. Functional Ecology, 21, 528–533. https://doi.org/10.1111/j.1365-2435.2007.01255.x

[ece33987-bib-0010] Burton, T. , Killen, S. , Armstrong, J. , & Metcalfe, N. (2011). What causes intraspecific variation in resting metabolic rate and what are its ecological consequences? Proceedings of the Royal Society B: Biological Sciences, 278, 3465–3473. https://doi.org/10.1098/rspb.2011.1778 21957133 10.1098/rspb.2011.1778 PMC3189380

[ece33987-bib-0011] Bushuev, A. , Husby, A. , Sternberg, H. , & Grinkov, V. (2012). Quantitative genetics of basal metabolic rate and body mass in free‐living pied flycatchers. Journal of Zoology, 288, 245–251. https://doi.org/10.1111/j.1469-7998.2012.00947.x

[ece33987-bib-0012] Chesson, P. (2000). Mechanisms of maintenance of species diversity. Annual Review of Ecology and Systematics, 31, 343–366. https://doi.org/10.1146/annurev.ecolsys.31.1.343

[ece33987-bib-0013] Chesson, P. , & Huntly, N. (1997). The roles of harsh and fluctuating conditions in the dynamics of ecological communities. The American Naturalist, 150, 519–553. https://doi.org/10.1086/286080 10.1086/286080 18811299

[ece33987-bib-0014] Chesson, P. L. , & Warner, R. R. (1981). Environmental variability promotes coexistence in lottery competitive systems. The American Naturalist, 117, 923–943. https://doi.org/10.1086/283778

[ece33987-bib-0015] Cramer, E. R. , Ålund, M. , McFarlane, S. E. , Johnsen, A. , & Qvarnström, A. (2016). Females discriminate against heterospecific sperm in a natural hybrid zone. Evolution, 70, 1844–1855. https://doi.org/10.1111/evo.12986 27312694 10.1111/evo.12986

[ece33987-bib-0016] Darwin, C . (1859). On the origin of species by means of natural selection, or, the preservation of favoured races in the struggle for life. PMC5184128 30164232

[ece33987-bib-0017] Forstmeier, W. , & Schielzeth, H. (2011). Cryptic multiple hypotheses testing in linear models: Overestimated effect sizes and the winner's curse. Behavioral Ecology and Sociobiology, 65, 47–55. https://doi.org/10.1007/s00265-010-1038-5 21297852 10.1007/s00265-010-1038-5 PMC3015194

[ece33987-bib-0018] Hadfield, J. D. , Heap, E. A. , Bayer, F. , Mittell, E. A. , & Crouch, N. (2013). Disentangling genetic and prenatal sources of familial resemblance across ontogeny in a wild passerine. Evolution, 67, 2701–2713. https://doi.org/10.1111/evo.12144 24033177 10.1111/evo.12144

[ece33987-bib-0019] Handelsman, C. A. , Broder, E. D. , Dalton, C. M. , Ruell, E. W. , Myrick, C. A. , Reznick, D. N. , & Ghalambor, C. K. (2013). Predator‐induced phenotypic plasticity in metabolism and rate of growth: Rapid adaptation to a novel environment. Integrative and Comparative Biology, 53, 975–988. https://doi.org/10.1093/icb/ict057 23784701 10.1093/icb/ict057

[ece33987-bib-0020] Keller, I. , & Seehausen, O. (2012). Thermal adaptation and ecological speciation. Molecular Ecology, 21, 782–799. https://doi.org/10.1111/j.1365-294X.2011.05397.x 22182048 10.1111/j.1365-294X.2011.05397.x

[ece33987-bib-0021] Kersten, M. , & Piersma, T. (1987). High levels of energy expenditure in shorebirds: Metabolic adaptations to an energetically expensive way of life. Ardea, 75, 175–187.

[ece33987-bib-0022] Klaassen, M. , Oltrogge, M. , & Trost, L. (2004). Basal metabolic rate, food intake, and body mass in cold‐and warm‐acclimated Garden Warblers. Comparative Biochemistry and Physiology Part A: Molecular & Integrative Physiology, 137, 639–647. https://doi.org/10.1016/j.cbpb.2003.12.004 10.1016/j.cbpb.2003.12.004 15123171

[ece33987-bib-0023] Kuznetsova, A. , Brockhoff, P. B. , & Christensen, R. H. B . (2014). lmerTest: Tests for random and fixed effects for linear mixed effects models (lmer objects of lme4 package). *R package version 2.0‐30*.

[ece33987-bib-0024] Lasiewski, R. C. , Hubbard, S. , & Moberly, W. (1964). Energetic relationships of a very small passerine bird. Condor, 66, 212–220. https://doi.org/10.2307/1365646

[ece33987-bib-0025] Lewden, A. , Petit, M. , & Vézina, F. (2012). Dominant black‐capped chickadees pay no maintenance energy costs for their wintering status and are not better at enduring cold than subordinate individuals. Journal of Comparative Physiology B, 182, 381–392. https://doi.org/10.1007/s00360-011-0625-8 10.1007/s00360-011-0625-8 22037961

[ece33987-bib-0026] Lighton, J. R. (2008). Measuring metabolic rates: A manual for scientists: A manual for scientists. Oxford, UK: Oxford University Press https://doi.org/10.1093/acprof:oso/9780195310610.001.0001

[ece33987-bib-0027] Lourdais, O. , Shine, R. , Bonnet, X. , Guillon, M. , & Naulleau, G. (2004). Climate affects embryonic development in a viviparous snake, *Vipera aspis* . Oikos, 104, 551–560. https://doi.org/10.1111/j.0030-1299.2004.12961.x

[ece33987-bib-0028] Lovegrove, B. (2003). The influence of climate on the basal metabolic rate of small mammals: A slow‐fast metabolic continuum. Journal of Comparative Physiology B, 173, 87–112. 10.1007/s00360-002-0309-5 12624648

[ece33987-bib-0029] Lundberg, A. , & Alatalo, R. V. (1992). The pied flycatcher. London, UK: T&AD Poyser.

[ece33987-bib-0030] McFarlane, S. E. , Sirkiä, P. M. , Ålund, M. , & Qvarnström, A. (2016). Hybrid Dysfunction Expressed as Elevated Metabolic Rate in Male Ficedula Flycatchers. PLoS One, 11, e0161547 https://doi.org/10.1371/journal.pone.0161547 27583553 10.1371/journal.pone.0161547 PMC5008804

[ece33987-bib-0031] McGill, B. J. , Enquist, B. J. , Weiher, E. , & Westoby, M. (2006). Rebuilding community ecology from functional traits. Trends in Ecology & Evolution, 21, 178–185. https://doi.org/10.1016/j.tree.2006.02.002 16701083 10.1016/j.tree.2006.02.002

[ece33987-bib-0032] McKechnie, A. E. (2008). Phenotypic flexibility in basal metabolic rate and the changing view of avian physiological diversity: A review. Journal of Comparative Physiology B, 178, 235–247. https://doi.org/10.1007/s00360-007-0218-8 10.1007/s00360-007-0218-8 17957373

[ece33987-bib-0033] Moreno, J. , & Carlson, A. (1989). Clutch size and the costs of incubation in the pied flycatcher *Ficedula hypoleuca* . Ornis Scandinavica, 20, 123–128. https://doi.org/10.2307/3676879

[ece33987-bib-0034] Moreno, J. , Cowie, R. J. , Sanz, J. J. , & Williams, R. S. (1995). Differential response by males and females to brood manipulations in the pied flycatcher: Energy expenditure and nestling diet. Journal of Animal Ecology, 64, 721–732. https://doi.org/10.2307/5851

[ece33987-bib-0035] Mueller, P. , & Diamond, J. (2001). Metabolic rate and environmental productivity: Well‐provisioned animals evolved to run and idle fast. Proceedings of the National Academy of Sciences, 98, 12550–12554. https://doi.org/10.1073/pnas.221456698 10.1073/pnas.221456698 PMC60091 11606744

[ece33987-bib-0036] Nadachowska‐Brzyska, K. , Burri, R. , Olason, P. I. , Kawakami, T. , Smeds, L. , & Ellegren, H. (2013). Demographic divergence history of pied flycatcher and collared flycatcher inferred from whole‐genome re‐sequencing data. PLoS Genetics, 9, e1003942 https://doi.org/10.1371/journal.pgen.1003942 24244198 10.1371/journal.pgen.1003942 PMC3820794

[ece33987-bib-0037] Nakagawa, S. , & Schielzeth, H. (2013). A general and simple method for obtaining R^2^ from generalized linear mixed‐effects models. Methods in Ecology and Evolution, 4, 133–142. https://doi.org/10.1111/j.2041-210x.2012.00261.x

[ece33987-bib-0038] Naya, D. E. , Spangenberg, L. , Naya, H. , & Bozinovic, F. (2013). Thermal conductance and basal metabolic rate are part of a coordinated system for heat transfer regulation. Proceedings of the Royal Society B: Biological Sciences, 280: 20131629 https://doi.org/10.1098/rspb.2013.1629 23902915 10.1098/rspb.2013.1629 PMC3735268

[ece33987-bib-0039] Nilsson, J. Å. , Åkesson, M. , & Nilsson, J. (2009). Heritability of resting metabolic rate in a wild population of blue tits. Journal of Evolutionary Biology, 22, 1867–1874. https://doi.org/10.1111/j.1420-9101.2009.01798.x 19682309 10.1111/j.1420-9101.2009.01798.x

[ece33987-bib-0040] Pigot, A. L. , & Tobias, J. A. (2013). Species interactions constrain geographic range expansion over evolutionary time. Ecology Letters, 16, 330–338. https://doi.org/10.1111/ele.12043 23231353 10.1111/ele.12043

[ece33987-bib-0041] Qvarnström, A. , Ålund, M. , McFarlane, S. E. , & Sirkiä, P. M. (2016). Climate adaptation and speciation: Particular focus on reproductive barriers in Ficedula flycatchers. Evolutionary Applications, 9, 119–134. https://doi.org/10.1111/eva.12276 27087843 10.1111/eva.12276 PMC4780377

[ece33987-bib-0042] Qvarnström, A. , Rice, A. M. , & Ellegren, H. (2010). Speciation in Ficedula flycatchers. Philosophical Transactions of the Royal Society B: Biological Sciences, 365, 1841–1852. https://doi.org/10.1098/rstb.2009.0306 10.1098/rstb.2009.0306 PMC2871891 20439285

[ece33987-bib-0043] Qvarnström, A. , Svedin, N. , Wiley, C. , Veen, T. , & Gustafsson, L. (2005). Cross‐fostering reveals seasonal changes in the relative fitness of two competing species of flycatchers. Biology Letters, 1, 68–71. https://doi.org/10.1098/rsbl.2004.0265 17148130 10.1098/rsbl.2004.0265 PMC1629061

[ece33987-bib-0044] Qvarnström, A. , Wiley, C. , Svedin, N. , & Vallin, N. (2009). Life‐history divergence facilitates regional coexistence of competing Ficedula flycatchers. Ecology, 90, 1948–1957. https://doi.org/10.1890/08-0494.1 19694142 10.1890/08-0494.1

[ece33987-bib-0045] R Core Team (2013). R: A language and environment for statistical computing. Vienna, Austria: R Foundation for Statistical Computing.

[ece33987-bib-0046] Rønning, B. , Jensen, H. , Moe, B. , & Bech, C. (2007). Basal metabolic rate: Heritability and genetic correlations with morphological traits in the zebra finch. Journal of Evolutionary Biology, 20, 1815–1822. https://doi.org/10.1111/j.1420-9101.2007.01384.x 17714299 10.1111/j.1420-9101.2007.01384.x

[ece33987-bib-0047] Rybinski, J. , Sirkiä, P. M. , McFarlane, S. E. , Vallin, N. , Wheatcroft, D. , Ålund, M. , & Qvarnström, A. (2016). Competition‐driven build‐up of habitat isolation and selection favoring modified dispersal patterns in a young avian hybrid zone. Evolution, 70, 2226–2238. https://doi.org/10.1111/evo.13019 27464950 10.1111/evo.13019

[ece33987-bib-0048] Sanz, J. J. (1997). Clutch size manipulation in the pied flycatcher: Effects on nestling growth, parental care and moult. Journal of Avian Biology, 28, 157–162. https://doi.org/10.2307/3677309

[ece33987-bib-0049] Schmidt‐Nielsen, K. (1997). Animal physiology: Adaptation and environment. New York, NY: Cambridge University Press.

[ece33987-bib-0050] Sirkiä, P. M. , McFarlane, S. E. , Jones, W. , Wheatcroft, D. , Ålund, M. , Rybinski, J. , & Qvarnström, A. (2017). Climate‐driven build‐up of temporal isolation within a recently formed avian hybrid zone. Evolution, 72, 363–374. 10.1111/evo.13404 29214649

[ece33987-bib-0051] Somero, G. (2010). The physiology of climate change: How potentials for acclimatization and genetic adaptation will determine ‘winners’ and ‘losers’. Journal of Experimental Biology, 213, 912–920. https://doi.org/10.1242/jeb.037473 20190116 10.1242/jeb.037473

[ece33987-bib-0052] Song, Z.‐G. , & Wang, D.‐H. (2006). Basal metabolic rate and organ size in Brandt's voles *Lasiopodomys brandtii*: Effects of photoperiod, temperature and diet quality. Physiology & Behavior, 89, 704–710. https://doi.org/10.1016/j.physbeh.2006.08.016 16989876 10.1016/j.physbeh.2006.08.016

[ece33987-bib-0053] Speakman, J. , & McQueenie, J. (1996). Limits to sustained metabolic rate: The link between food intake, basal metabolic rate, and morphology in reproducing mice, Mus musculus. Physiological Zoology, 69, 746–769. https://doi.org/10.1086/physzool.69.4.30164228

[ece33987-bib-0054] Spicer, J. , & Gaston, K. (2009). Physiological diversity: Ecological implications. Hoboken NJ: John Wiley & Sons.

[ece33987-bib-0055] Stager, M. , Pollock, H. S. , Benham, P. M. , Sly, N. D. , Brawn, J. D. , & Cheviron, Z. A. (2015). Disentangling environmental drivers of metabolic flexibility in birds: The importance of temperature extremes versus temperature variability. Ecography, 39, 787–795.

[ece33987-bib-0056] Stenseth, N. C. , Mysterud, A. , Ottersen, G. , Hurrell, J. W. , Chan, K.‐S. , & Lima, M. (2002). Ecological effects of climate fluctuations. Science, 297, 1292–1296. https://doi.org/10.1126/science.1071281 12193777 10.1126/science.1071281

[ece33987-bib-0057] Swanson, D. L. , McKechnie, A. E. , & Vézina, F. (2017). How low can you go? An adaptive energetic framework for interpreting basal metabolic rate variation in endotherms. Journal of Comparative Physiology B, 187, 1039–1056. https://doi.org/10.1007/s00360-017-1096-3 10.1007/s00360-017-1096-3 28401293

[ece33987-bib-0058] Tayleur, C. , Caplat, P. , Massimino, D. , Johnston, A. , Jonzén, N. , Smith, H. G. , & Lindström, Å. (2015). Swedish birds are tracking temperature but not rainfall: Evidence from a decade of abundance changes. Global Ecology and Biogeography, 24, 859–872. https://doi.org/10.1111/geb.12308

[ece33987-bib-0059] Vallin, N. , Rice, A. M. , Arntsen, H. , Kulma, K. , & Qvarnström, A. (2012). Combined effects of interspecific competition and hybridization impede local coexistence of Ficedula flycatchers. Evolutionary Ecology, 26, 927–942. https://doi.org/10.1007/s10682-011-9536-0

[ece33987-bib-0060] Versteegh, M. A. , Helm, B. , Dingemanse, N. J. , & Tieleman, B. I. (2008). Repeatability and individual correlates of basal metabolic rate and total evaporative water loss in birds: A case study in European stonechats. Comparative Biochemistry and Physiology Part A: Molecular & Integrative Physiology, 150, 452–457. https://doi.org/10.1016/j.cbpa.2008.05.006 10.1016/j.cbpa.2008.05.006 18571446

[ece33987-bib-0061] Visser, M. , Van Noordwijk, A. , Tinbergen, J. , & Lessells, C . (1998). Warmer springs lead to mistimed reproduction in great tits (*Parus major*). Proceedings of the Royal Society of London. Series B: Biological Sciences, 265, 1867–1870. https://doi.org/10.1098/rspb.1998.0514

[ece33987-bib-0062] Wallace, A. R. (1878). Tropical nature, and other essays. London, UK: Macmillan and Company https://doi.org/10.5962/bhl.title.1261

[ece33987-bib-0063] Wiley, C. , Fogelberg, N. , Sæther, S. A. , Veen, T. , Svedin, N. , Kehlenbeck, J. V. , & Qvarnström, A. (2007). Direct benefits and costs for hybridizing Ficedula flycatchers. Journal of Evolutionary Biology, 20, 854–864. https://doi.org/10.1111/j.1420-9101.2007.01316.x 17465896 10.1111/j.1420-9101.2007.01316.x

[ece33987-bib-0064] Zub, K. , Borowski, Z. , Szafrańska, P. A. , Wieczorek, M. , & Konarzewski, M. (2014). Lower body mass and higher metabolic rate enhance winter survival in root voles, *Microtus oeconomus* . Biological Journal of the Linnean Society, 113, 297–309. https://doi.org/10.1111/bij.12306

